# Glucosamine Phosphate Induces AuNPs Aggregation and Fusion into Easily Functionalizable Nanowires

**DOI:** 10.3390/nano9040622

**Published:** 2019-04-17

**Authors:** Álvaro Martínez, Yanchao Lyu, Fabrizio Mancin, Paolo Scrimin

**Affiliations:** 1 Department of Chemical Sciences, University of Padova, via Marzolo, 1, 35131 Padova, Italy; alvaro.martinez@dipc.org (Á.M.); yanchao.lyu@studenti.unipd.it (Y.L.); fabrizio.mancin@unipd.it (F.M.); 2 Donostia International Physics Center, Paseo Manuel de Lardizabal 4, 20018 Donostia, Spain

**Keywords:** gold nanoparticles, glucosamine phosphate, gold nanowires, surface plasmon resonance

## Abstract

The challenge to obtain plasmonic nanosystems absorbing light in the near infrared is always open because of the interest that such systems pose in applications such as nanotherapy or nanodiagnostics. Here we describe the synthesis in an aqueous solution devoid of any surfactant of Au-nanowires of controlled length and reasonably narrow dimensional distribution starting from Au-nanoparticles by taking advantage of the properties of glucosamine phosphate under aerobic conditions and substoichiometric nanoparticle passivation. Oxygen is required to enable the process where glucosamine phosphate is oxidized to glucosaminic acid phosphate and H_2_O_2_ is produced. The process leading to the nanosystems comprises nanoparticles growth, their aggregation into necklace-like aggregates, and final fusion into nanowires. The fusion requires the consumption of H_2_O_2_. The nanowires can be passivated with an organic thiol, lyophilized, and resuspended in water without losing their dimensional and optical properties. The position of the broad surface plasmon band of the nanowires can be tuned from 630 to >1350 nm.

## 1. Introduction

The aggregation of gold nanoparticles (AuNPs) into clusters is a well-known phenomenon that can be induced by crosslinking agents, cationic surfactants, or salts. In this latter case, Derjaguin, Landau, Verwey, and Overbeek (DLVO) suggested that the interactions between particles are governed by superposition of van der Waals forces and double layer forces [1,2]. It has been shown that aggregation occurs more easily in organic solvents than in water [3] because of the higher solvation power of pure water to ions. Typical crosslinking agents are molecules with at least two functional groups able to interact with the gold surface. The most popular ones are bisthiols [4,5]. For naked gold nanoparticles, i.e., those devoid of passivating molecules or with their surface not fully passivated, aggregation can be a slow, spontaneous process that eventually leads to the precipitation of the clusters formed. It has been reported that the shape (globular or linear) of the aggregates formed depends on whether the aggregation process is a diffusion- or reaction-limited process [6,7]. Conditions for forming linear, necklace-like [8,9] or globular [10] aggregates have been reported. The solvent used [4,11] passivating units, or the addition of salts [3,12] to influence the process leading, prevailingly, to one or the other aggregate morphology. Aggregation strongly affects the spectroscopic properties of AuNPs. For this reason, spectrophotometry is of great help in assessing the morphology of the aggregate formed [13]. A necklace-like aggregate formation is highlighted by the development of an additional, new surface plasmon resonance (SPR) band at 650–750 nm, red-shifted with respect to the 520–525 nm band of the single, isolated AuNP. On the contrary, close-packed, globular aggregates exhibit a single band at *ca*. 550–570 nm.

For several important applications like, for instance, surface-enhanced Raman scattering (SERS) for diagnostic purposes [14,15] or thermal therapy in nanomedicine [16,17] it would be desirable to further red-shift the new band formed with the necklace-like aggregate formation because tissues absorb less in the near infrared (NIR) region. The extent of the shift depends on the length of the aggregate and, very importantly, on the distance between the nanoparticles, the closer they are the more red-shifted is the band [18]. The shift of the plasmon resonance band beyond 700–750 nm can only be obtained by fusing the nanoparticles into a nanowire. [19,20] It is well known that the plasmon band of gold nanorods red-shifts with the increase of the aspect ratio [21]. In fact, nanowires (NWs), depending on their length, absorb well within the NIR region [16,22]. Most of the several “wet” conditions for the preparation of NWs use amphiphilic molecules like oleylamine [23,24] or cetyltrimethylamminium bromide [25]. For biological applications, these protocols pose a serious problem of contamination of the nanostructure by the toxic cationic additives used for their preparation [26]. Fusion of linear aggregates by laser irradiation and not by chemical reaction has also been reported [16,27]. We report here the synthesis of gold nanowires under milder conditions. Our results are based on the serendipitous observation that the addition of glucosamine phosphate (GAP) to AuNPs led to the growth and fusion of them into nanowires. We were actually looking for a mild passivating agent to replace citrate [28] in AuNPs. However, the presence of the glucose moiety on GAP, as we will show below, resulted in a cascade of redox processes [29] starting with AuNPs aggregation in a linear fashion and ending with their fusion into nanowires.

## 2. Materials and Methods

### 2.1. Chemicals and Instruments

GAP, compound **1**, tris(hydroxymethyl)aminomethane (TRIS), H_2_O_2_, and solvents were obtained from Sigma-Aldrich (Merck KGaA, Darmstadt, Germany) and used as received without further purification unless stated otherwise. Zwitterionic thiol **2** was prepared by following previously reported procedures [30].

Glassware in contact with gold nanoparticles was washed with *aqua regia* before and after its use and rinsed with distilled water. All gold nanoparticle preparations and purifications were carried out with milliQ water. Nanoparticles were purified by centrifugation on a Hettich Universal 320 R centrifuge (Andreas Hettich GmbH & Co.KG, Tuttlingen, Germany) operating with a swinging rotor (*V* ≤ 15 mL, rpm ≤ 5000), a 45° fixed angle rotor (*V* ≤ 5 mL, rpm ≤ 12,000) or an Eppendorf miniSpin Plus (Eppendorf AG, Hamburg, Germany) (*V* ≤ 1.5 mL, rpm ≤ 14,500) depending on the sample volume.

UV–Visible spectra were acquired on a Varian Cary 50 or Cary 100 spectrophotometer (Agilent Technologies, Santa Clara, CA, USA) whereas UV–Vis–NIR spectra were acquired on a Varian Cary 5000 spectrophotometer (Agilent Technologies, Santa Clara, CA, USA) employing 10 mm path length Hellma Suprasil^®^ quartz cuvettes.

Transmission electron microscopy (TEM) analyses were run on a FEI Tecnai G12 microscope (Thermo Fisher Scientific, Hillsboro, OR, USA) operating at 100 kV and images registered with an OSIS Veleta 4 K camera (Olympus Soft Imaging Solutions GmbH, Münster, Germany). Samples were typically deposited on a copper grid and the excess of solvent removed with filtering paper. Size distribution analysis was carried out by modelling nanoparticle intensity profiles employing Pebbles (v2.0.1, A. Ponti et al., ISTM, CNR, Rome, Italy) and size distribution calculated by performing direct statistics on the previously modelled nanoparticles with PebbleJuggler software (v1.0, A. Ponti et al., ISTM, CNR, Rome, Italy) [31]. Nanowire length and width were measured with ImageJ (v1.51, W. Rasband et al., NIH, Bethesda, MD, USA); length measurements were performed by modelling the nanowires as a series of straight linear segments.

### 2.2. Gold Nanoparticles Preparation

A modified version of a literature procedure was followed [32]. Typically, to 5.6 mL of water, sequentially and under vigorous stirring, 1.6 mL of sodium citrate (510 mM), 250 µL of silver nitrate (10 mM), and 500 µL of tetrachloroauric acid (250 mM) were added and stirred for 5 min. During this time, the solution changed from initial yellow color to green, grey, and finally black. After the incubation time, the solution was quickly added to 117 mL of boiling water and heated under reflux for 1 h, becoming wine-red after a few seconds. The citrate-capped nanoparticles solution obtained was then allowed to cool down to room temperature before addition of GAP.

### 2.3. Citrate Depletion

To the above AuNPs solution (125 mL), GAP (8 mg), dissolved in 1 mL of water, was added and the solution stirred for 10 min. The final concentration of GAP was, accordingly, 25 µM. Free molecules in solution were removed by using 15 or 4 mL Amicon^®^ Ultra filters of 100 KDa molecular weight cutoff centrifuged for 2.5 min at 2000 rpm. Prior to use, filters were prewashed twice with 1:1 H_2_O:MeOH and 3 times with H_2_O, then nanoparticles were washed 5 times with water. Typical concentrated volumes after centrifugation for 15 and 4 mL were 1.5 and 0.4 mL respectively. To determine the amount of organic material present before and after the depletion protocol we carried out a thermogravimetric analysis (TGA) of the two AuNPs preparations [33,34] (see Figure S1 and Table S1 of Supplementary Materials).

### 2.4. Necklaces and Nanowire Formation in H_2_O

Aggregation and nanowire formation processes were typically followed by using approximately 100 µL of the above centrifuged sample, diluted up to 3 mL and placed in a cuvette. Temperature was kept constant and equal to 30 °C during all experiments. In addition to the experiments done in the cuvettes, nanowires have also been isolated after functionalization (passivation) with **2**. By employing 60 mL of as-prepared AuNPs solution a final amount of 6.6 mg of NWs was obtained (approx. yield based on Au, 50%).

### 2.5. Oxygen Depletion

A nitrogen flow was applied to the as-prepared AuNPs for 1 h. Nanoparticles were then filtered as usual and the resulting cuvette, after dilution, was deoxygenated again by bubbling with nitrogen for an additional 10 min. Cuvettes were sealed with parafilm and samples incubated as usual.

### 2.6. XPS Analysis

X-ray photoelectron spectroscopy (XPS) spectra were collected in an Ultra High Vacuum chamber equipped with an EA 125 Omicrom electron analyzer (Scienta Omicron GmbH, Taunusstein, Germany). Core level photoemission spectra (C 1s, O 1s, N 1s, Ag 3d, and Au 4f regions) were collected at room temperature in normal emission with a non-monochromatized Al Kα X-ray source (hν = 1486.6 eV) using 0.1 eV steps, 0.5 s collection time, and 20 eV pass energy. The analyzed sample was prepared by drop casting the water solution containing Au NPs on a copper substrate. After drying in air, the obtained film was introduced into the ultrahigh vacuum chamber and de-gassed overnight. The XPS photoemission lines were separated into individual components (after Shirley background removal) using symmetrical Voigt functions and nonlinear least-squares routines for the χ^2^ minimization. The results are reported in the Supplementary Materials (Figure S2 and Table S2).

## 3. Results and Discussion

**Figure 1:**
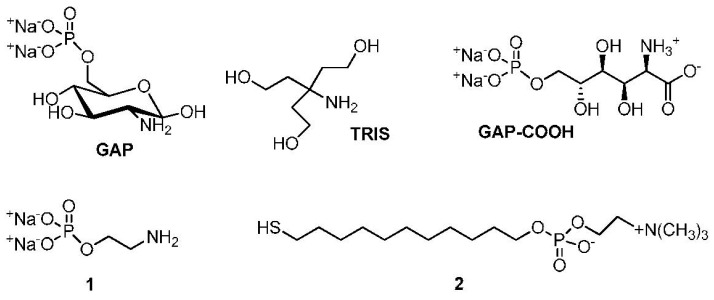
Compounds discussed in this work.

Our original idea was to find a mild capping agent for AuNPs that could allow them to remain stable in solution without aggregation even when used in stoichiometric concentration to passivate the nanoparticle surface. Glucosamine phosphate (GAP, Figure 1) proved suitable for this purpose. Indeed, under these conditions (i.e., [GAP] ≥ [Au_surface_]) AuNPs are stable as can be judged by the intensity and position of the SPR band monitored over several days. Phosphonic acid derivatives bearing an amino group like aminomethylene phosphonic acid are mild passivating agents of AuNPs [35] and GAP behaves in a similar way. It appears to be able to passivate AuNPs by interacting with the gold surface more strongly than citrate because of the presence of the amino group [36]. However, when GAP is used under substoichiometric concentrations, i.e., at a concentration lower than that of the free Au atoms present on the surface of AuNPs, a slow aggregation process of the nanoparticles occurs that evolves into the formation of nanowires. As we discuss below the glucose moiety of GAP is necessary for the final outcome of the process. Oxidation of glucose to gluconic acid under aerobic conditions in the presence of AuNPs was first reported by Rossi et al. [37]. They observed a steady growth of nanoparticles during the process. It was later discovered [38] that hydrogen peroxide was also formed in addition to gluconic acid [29,39]. Hydrogen peroxide is known to be able to reduce Au(III) and Au(I) to Au(0) in the presence of the AuNPs [29]. All the above processes are involved in the formation of nanowires from AuNPs as we discuss here.

### 3.1. Preparation of the AuNPs

**Figure 2:**
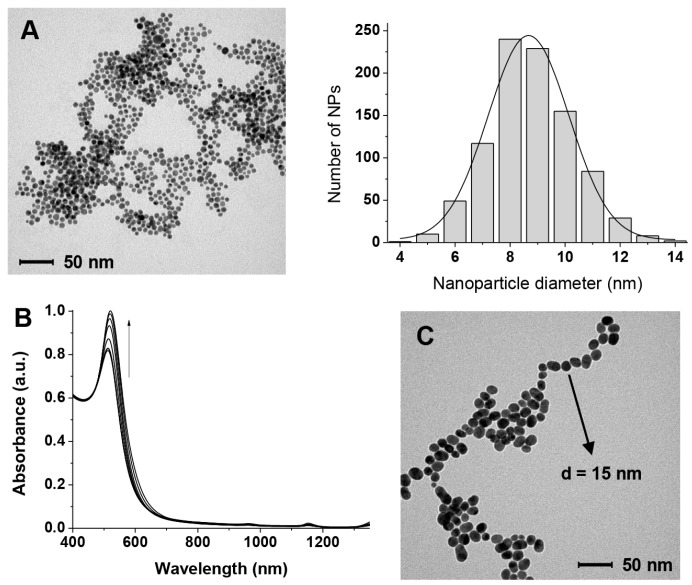
(**A**) TEM image and size distribution of the gold nanoparticles (AuNPs) used in this work passivated with citrate; (**B**) Evolution with time of the Vis spectrum in the plasmon resonance region of the AuNPs after citrate depletion (over 7 days, curves were recorded at 1-day intervals); (**C**) TEM image of the citrate-depleted AuNPs after 7 days (notice the same scale bar as in (A)).

The AuNPs were prepared as described in Section 2.2 by reduction of Au(III) with citrate. Their size was estimated by TEM (Figure 2A) to be 8.5 ± 1.5 nm. The washing procedures with water (Section 2.3) resulted in the depletion of most of the citrate. TGA reveals that the percentage of organic material is reduced from 14.4% to 4.3% after the washing cycles. By considering 8.5 nm spherical nanoparticles (the spherical shape is an acceptable simplification for nanoparticles of this size) [33] we estimated, as discussed elsewhere [34] that *ca*. 30% of the gold atoms on the surface of the nanoparticles is not passivated in the “citrate-depleted” nanoparticles compared to none before the organic material removal protocol we performed. The Zeta potential of the as-prepared AuNPs is −25.5 mV while that of the citrate-depleted ones drops to −2.5 mV. The TGA experiments indicate that, when GAP is added, the amount of organic material remaining after the depletion process is higher (see Figure S1 of Supplementary Materials). Our estimate is that [GAP]_final_ is 4–5 µM.

### 3.2. GAP-Induced Aggregation in Water and NWs Formation

The AuNPs we prepared are stable under the preparation conditions ([sodium citrate] = 6.8 × 10^−3^ M) for prolonged times. However, when most of the citrate is removed (citrate-depleted AuNPS, see Section 2.3) they start very slowly to cluster [40,41] as can be seen in the absorbance spectrum (slight increase of absorbance >600 nm in Figure 2B) and increase in size (increase in absorbance at 525 nm in Figure 2B and TEM image in Figure 2C). Clearly, under these low passivation conditions, Ostwald ripening cannot be prevented.

**Figure 3:**
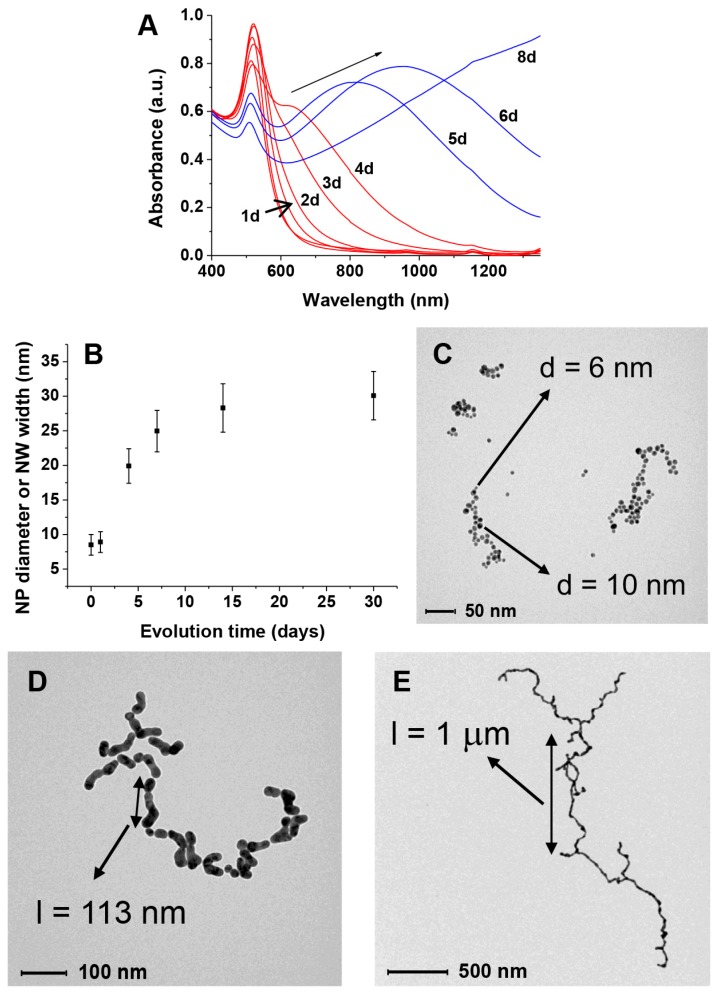
Behavior of citrate-depleted AuNPs in the presence of GAP: (**A**) evolution of the UV–Vis spectrum with time. In red, necklace formation, in blue, nanoparticle fusion; (**B**) increase in size of the AuNPs or nanowires width with time; (B–D) TEM spectra taken at increasing times (**C**) 1day; (**D**) 4 days; (**E**) 8 days.

**Figure 4:**
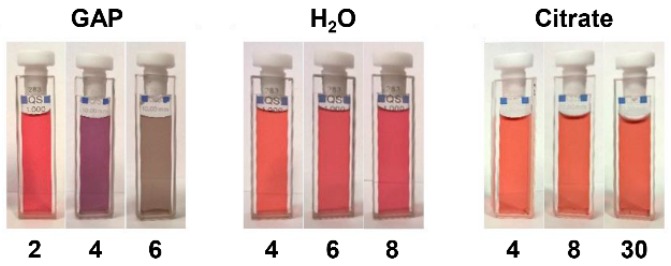
Change of color of the different AuNPs preparations with time. Timescale in days.

On the other hand, if GAP is present in the “citrate depleted” AuNPs we observe not only the growth in size of the nanoparticles (Figure 3B) but also a relatively faster aggregation process (from days to hours) leading to necklace-like aggregates as revealed by the formation of an additional absorption band at 650–680 nm (Figure 3A, traces in red). This aggregation process only occurs when substoichiometric amounts of GAP are added. After longer times (days) this band broadens and shifts to longer wavelengths up to >1200 nm (Figure 3A, traces in blue). Throughout the experiment, the solution pH remains constant at 6.5. TEM images taken at different time intervals revealed the formation of nanowires of increasing length resulting from the fusion of the formed necklace-like aggregates (Figure 3C–E). The aggregation and fusion processes can be also followed by the naked eye as can be seen in Figure 4. As a matter of fact, the two processes are not fully separated and some fusion already occurs after four days.

**Figure 5:**
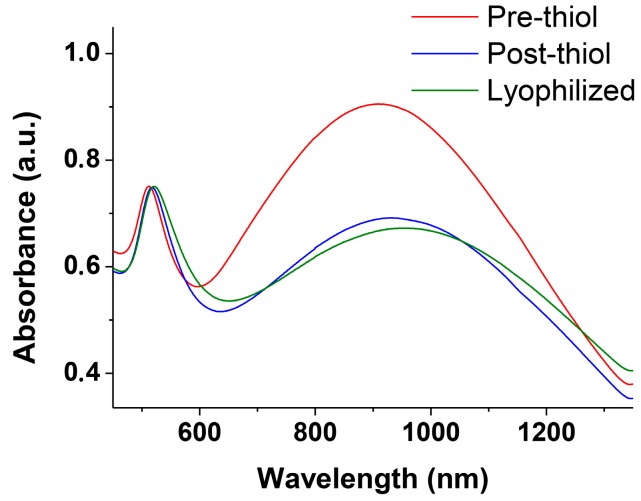
Vis–NIR spectra in the plasmon resonance region of nanowires before passivation with thiol **2**, immediately after the passivation with thiol **2** and after lyophilization and redissolution in water.

**Figure 6:**
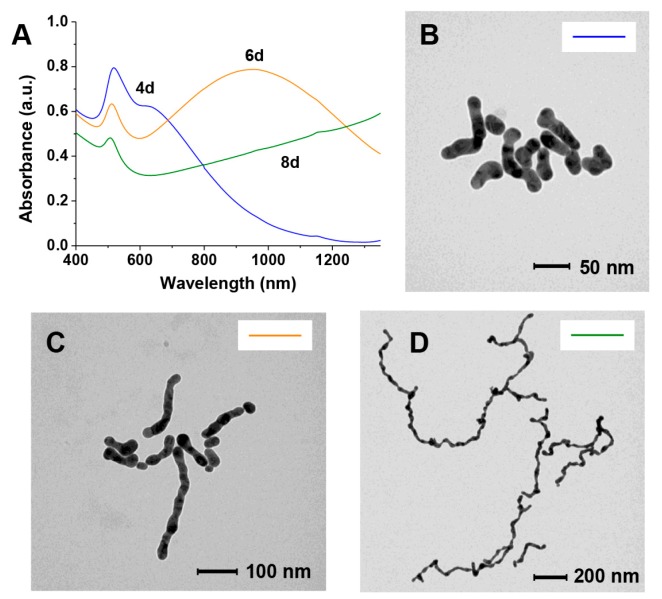
Absorption spectra (**A**) and TEM images of samples collected after (**B**) 4 days, (**C**) 6 days, or (**D**) 8 days and passivated with thiol **2**. The color of the line in the inset in each TEM image links it to the corresponding UV–Vis spectrum in A.

Very interestingly, if aliquots of the solution are collected at different time intervals and treated with thiol **2** (Figure 1) the obtained NWs are “frozen” and prevented from any further growth. These passivated nanowires can be lyophilized and redissolved showing a spectrum identical to the one recorded at the time of the thiol addition. The only relevant difference is a decrease of the intensity of the longer wavelength band in part associated with the passivation process (Figure 5). TEM analysis of these samples (Figure 6) reveals NWs characterized by a broad-length distribution. The average aspect ratio of such nanowires increases with the time allowed for the original AuNPs to evolve, prior to the addition of thiol **2** (Table 1). At variance with nanorods that show relatively narrow SPR bands depending on their aspect ratio, our nanowires present rather broad SPR. This is the result of the coexistence of several plasmon modes related to the broad distribution of their length and the existence of branching points (clearly visible in Figure 3D,E) [16].

### 3.3. Analysis of the Different Processes Occurring and the Role of GAP

#### 3.3.1. The Role of the Functional Groups of GAP in the Aggregation of AuNPs under Substoichiometric Passivation Conditions

The experimental results indicate that the aggregation of the AuNPs only occurs when the surface Au atoms are not fully passivated either by citrate or GAP. Our estimate (see Section 2.3) is that *ca*. 5 µM GAP is still present in the “citrate depleted” AuNPs. We calculated (see Section 2 in Supplementary Materials) that the concentration of the surface Au atoms of these 8.5 nm AuNPs is *ca*. 30 μM. This means that the amount of GAP is *ca*. 18% of that required to fully saturate the nanoparticles surface. Incidentally, this GAP concentration is also the optimum one required for crosslinking of similarly citrate-depleted AuNPs by using amino acids [34]. The group with the strongest affinity for AuNPs among those present on GAP is the primary amine. Passivation of AuNPs by amines, although leading to less robust nanoparticles than those passivated with thiols, is well documented [42,43,44]. What is the other functional group responsible for the crosslinking? Those present on GAP, apart from the amine, are the sugar hydroxyls and the phosphate groups. *O*-phosphorylethanolamine (**1**, Figure 1) is an amino phosphate devoid of the sugar moiety. When GAP is replaced by **1**, AuNPs grow very little and do not aggregate at all (see Figure S3 of Supplementary Materials). Tris(hydroxymethyl)aminomethane (TRIS, Figure 1) is an amino-alcohol [13]. It induces very limited linear aggregation towards necklaces that do not evolve into nanowires (see Figure S3 of Supplementary Materials). Accordingly, neither the hydroxyls nor the phosphate present on GAP appear to be involved in the crosslinking process. As is shown below, a new functional group is formed during the early steps of the overall process: a carboxylate. This is responsible for the aggregation in a process not much different from that observed with some amino acids [34].

#### 3.3.2. The Role of Glucosamine

All experimental evidence points to a critical role played by the glucose subunit present in GAP. It is known that glucose is involved in several redox process in the presence of Au(I) or Au(III) ions and AuNPs, as well. It is able to reduce HAuCl_4_ into AuNPs [37]. The AuNPs, once formed, oxidize glucose to gluconic acid while reducing O_2_ to H_2_O_2_ [28,38,39]. Furthermore, H_2_O_2_ is also able to reduce AuCl_4_^−^ to Au(0) [45,46,47]. The oxidation of GAP was demonstrated in our case by analyzing the organic component of AuNPs passivated with an excess of GAP. Under these conditions, obviously, the AuNPs do not crosslink but the amount of GAP is such to allow its quantification with time. ^1^H-NMR analysis of the organic component of the AuNPs after several days reveals (see Figure S4 of Supplementary Materials) the disappearance of the signals amenable to GAP and the appearance of signals pertaining to the oxidized glucosaminic acid phosphate (GAP-COOH, Figure 1) derivative. Thus, during this time, GAP is oxidized to GAP-COOH. Depletion of O_2_ from the system prevents the oxidation. In the absence of O_2,_ GAP behaves in the very same way as **1** does (see Figure S5 of Supplementary Materials) and aggregation is not observed. The above results indicate that GAP-COOH, at low concentration, is responsible for the linear aggregation of the nanoparticles. This implies that a carboxylate group has a higher affinity for the nanoparticle surface than a phosphate group. The aggregation is hence indirectly initiated by a redox process requiring both the glucose moiety of GAP and O_2_.

#### 3.3.3. The Overall Process

The overall process of formation of the nanowires requires the following steps: (a) the AuNPs growth and oxidation of GAP to GAP-COOH; (b) the formation of necklace-like aggregates; (c) the fusion of the aggregates into nanowires.

**Figure 7:**
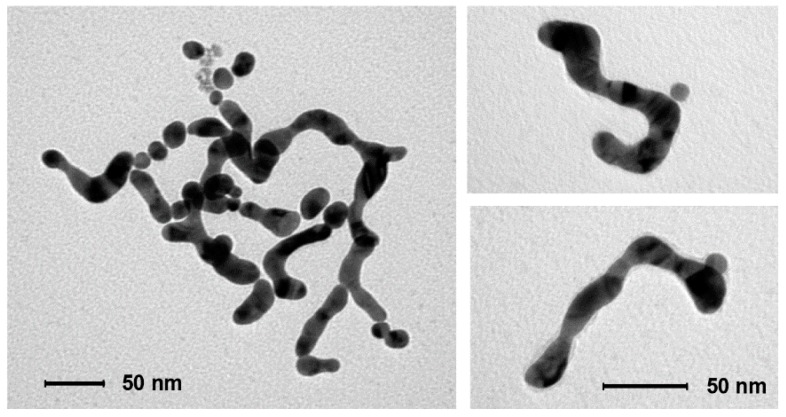
TEM image of the material collected from a solution of AuNPs after NaCl-induced linear aggregation and subsequent addition of H_2_O_2_. Nanowires were obtained without adding GAP.

The initial growth of the original nanoparticles (Figure 3B) is a well-known phenomenon reported for poorly-passivated gold nanoparticles [48]. AuNPs growth leads to the decrease of their overall surface area and, hence, the amount of passivating GAP and GAP-COOH required for their stabilization is also lower. Furthermore, larger nanoparticles are less prone to aggregation and subsequent coalescence [48]. Both these points explain why the growth of the nanoparticles, which reflects on the width of the final nanowires, stops at *ca*. 25 nm from the 8.5 nm diameter of the original ones. We observe that nanoparticles growth is fast at the early stages of the process while it slows down considerably with time (Figure 3B). However, the key question is what leads to nanoparticle fusion once the linear aggregates are formed. It has been reported that 3–5 nm AuNPs prepared by citrate reduction of HAuCl_4_ followed by further reduction with NaBH_4_ still contain *ca*. 4% Au(I) [49]. In the absence of the final NaBH_4_ reduction, as in our case, the amount is expected to be larger. XPS analysis of our as-prepared-AuNPs reveals that *ca*. 9–10% Au(I) is still present (Figure S2 and Table S2 of SM). Cold welding of ultrathin gold nanowires has been reported as the result of fast surface-atom diffusion under low pressure [50]. Such atom diffusion has been also suggested in the case of NWs formation in the presence of surfactants [25]. We hypothesize, however, that the reduction of residual Au(I) by the H_2_O_2_ produced in the reduction of O_2_ could provide the “glue” for fusing the AuNPs together when the necklaces are already formed. To test this hypothesis we prepared linear AuNPs aggregates by addition of NaCl in EtOH following a reported procedure [3]. Their aggregation is reversible, as reported. However after the addition of H_2_O_2_, rapid, irreversible fusion is observed as shown in Figure 7. This strongly supports the suggestion that H_2_O_2_ reduces the residual Au(I) present in the aggregated nanoparticles leading to their fusion into NWs.

**Figure 8:**
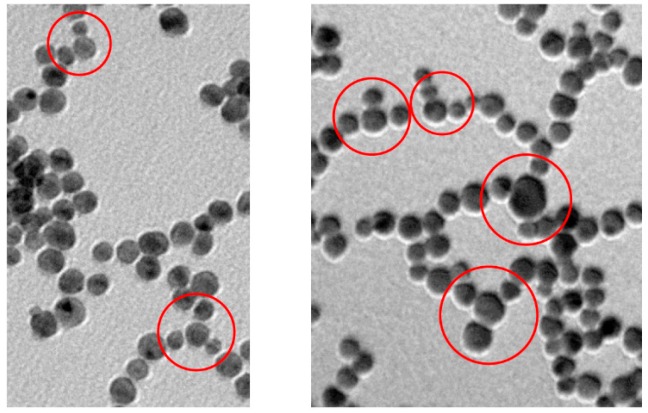
TEM pictures with highlighted branching points formed due to the non-linear disposition of AuNPs of small size in two different samples still mostly at the necklace-like aggregation state.

When we examine in detail the formed nanowires, the “memory” of the original nanoparticles is manifested by their wavy aspect, which is not much different from that observed by Xia et al. [25]. for nanowires prepared in the presence of cetyl-trimethylammonium bromide. We cannot rule out that Au atoms could diffuse along individual nanowires to generate smooth surfaces, but this is not the process that starts the AuNPs fusion. Contrary to ultrathin NWs [24,51,52] ours are very likely polycrystalline rather than single-crystal structures. The branching of the nanowires occasionally observed could be due to the merging of smaller diameter nanoparticles. It has been demonstrated that, while gold nanoparticles of the same size aggregate in a linear fashion, the coexisting smaller ones are less selective leading to lateral aggregation (Figure 8) [8]. Obviously, during the growth process, nanoparticles of different size coexist in spite of the rather narrow size distribution of the original AuNPs preparation. The wavy morphology of these NWs and the absence of any surfactant for their preparation is likely on the basis of their easy passivation, in strong contrast with what is typically observed with gold nanorods for which ligand exchange is not a trivial endeavor [53].

To sum up, the experimental evidence suggests that the small amount of GAP present after the partial depletion process is oxidized to GAP-COOH in the presence of O_2_, which, in turn is reduced to H_2_O_2_. GAP-COOH is responsible for the aggregation of AuNPs into mostly necklace-like aggregates while the reduction of residual Au(I) to Au(0) by H_2_O_2_ is responsible for the fusion of the aggregates with formation of nanowires. It has been reported [54] that gold nanorods can be oxidized by O_2_ to Au(I) under acidic conditions and high temperature in the presence of CTAB leading to their shortening. Although our conditions are much different from those reported for such a process to occur, we cannot rule out that a similar oxidation reaction could constitute an additional source of Au(I) for the fusion of our AuNPs.

## 4. Conclusions

**Figure 9:**
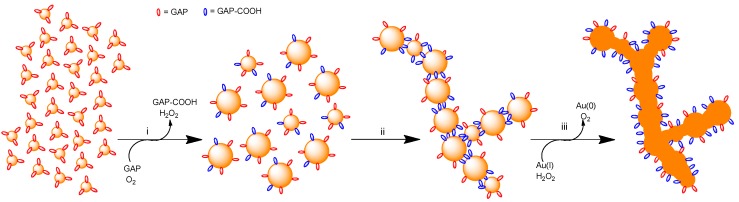
Cartoon rendition of AuNPs evolution towards nanowires: (**i**) GAP (red) poorly-passivated, small AuNPs grow by clustering and coalescence; the nanoparticles catalyze the oxidation of GAP to GAP-COOH (blue) and O_2_ is reduced to H_2_O_2_; (**ii**) larger nanoparticles aggregate to form necklaces in a process mostly driven by GAP-COOH. Ramifications occurs because of the less selective aggregation of smaller nanoparticles; (**iii**) residual Au(I) is reduced to Au(0) by H_2_O_2_ and this induces fusion and nanowires formation.

We report here a straightforward and mild procedure to induce the aggregation of AuNPs mostly in a linear fashion to form necklaces that eventually fuse into nanowires. Experimental evidence indicates that the evolution of the nanoparticles into nanowires is associated with a redox process catalyzed by the nanoparticles involving the oxidation of the glucose moiety of GAP, the reduction of O_2_ to H_2_O_2_ and eventually, the reduction of remaining Au(I) ions present in the gold clusters by the H_2_O_2_ formed. The product of the oxidation of GAP, the gluconic acid derivative GAP-COOH, appears to drive the mostly linear aggregation of the AuNPs while their fusion requires the reduction of residual Au(I) to Au(0) by H_2_O_2_. Because of the small amount of passivating agent present at the onset of the experiments, nanoparticles grow quickly by interacting together and coalescing into bigger ones. At the early stages of the process, GAP exerts the double role of a reducing agent on one side and a source of the efficient crosslinking compound GAP-COOH on the other. Fusion of the linear aggregates into nanowires is then due to reduction of residual Au(I) by H_2_O_2_. The overall process is depicted in Figure 9. The final result is the synthesis of NWs with a broad SPR band centered at a wavelength that is more red-shifted the longer the incubation time. They can reach wavelengths well above 1000 nm. These nanowires can be covered with a thiol that, by forming a surrounding, passivating monolayer, stabilizes them, prevents any further growth, and allows their lyophilization and resuspension in water without any significant change in optical properties.

We believe that our results are very important for several applications, particularly in the field of nanomedicine [55] in view of the great interest in nanosystems presenting plasmon resonance bands shifted in the IR region where cells and tissues do not absorb the radiation. Accordingly, also because of the use of non-toxic GAP in water and without any organic solvent or surfactant [56] and the ease of functionalization, they can be addressed for therapeutically relevant or analytical purposes.

## Figures and Tables

**Table 1:** Dimensions of the nanowires after stopping the AuNPs aggregation and fusion at different times and the maximum of the longer-wavelength plasmon resonance band.

Length/nm ^a^	Width/nm ^a^	Aspect Ratio ^a^	λ_max_
59 ± 29	21 ± 3	3 ± 1	634 nm
269 ± 112	24 ± 3	11 ± 4	952 nm
988 ± 212	26 ± 4	38 ± 12	>1350 nm

^a^ Average of 17 (634 nm), 12 (952 nm) or 7 (>1350 nm) nanowires from 2–3 different TEM pictures.

## Supplementary Materials

The following are available online at https://www.mdpi.com/2079-4991/9/4/622/s1, Figure S1: Thermogravimetric analysis of the citrate-passivated AuNPs, Table S1: Thermogravimetric analysis of nanoparticles, Figure S2: Au 4f photoemission line, Table S2: Surface elemental composition, Figure S3: Effect of addition of **2** or TRIS to AuNPs, Figure S4: ^1^H-NMR spectra of GAP in the presence of AuNPs after 2 weeks, Figure S5: UV-Vis spectra of AuNPs treated with GAP in the absence of O_2_.
