# Supplementary material for: Glucosamine Phosphate Induces AuNPs Aggregation and Fusion into Easily Functionalizable Nanowires
Source: Nanomaterials (Basel). 2019 Apr 17;9(4):622. doi: 10.3390/nano9040622 (PMC6523341; doi:10.3390/nano9040622)
Supplement: Supplementary file 1 [file nanomaterials-09-00622-s001.pdf]

Supplementary Materials

for

Glucosamine Phosphate Induces AuNPs Aggregation and Fusion into Easily Functionalizable Nanowires

Álvaro Martínez,[a,b] Yanchao Lyu,[a] Fabrizio Mancin,[a] and Paolo Scrimin[a,]*

[a]*Department of Chemical Sciences, University of Padova, via Marzolo, 1, 35131 Padova, Italy*
[b] *Donostia International Physics Center. Paseo Manuel de Lardizabal 4, Donostia, 20018, Spain*

Content:

1. Thermogravimetric analyses of three different samples (citrate passivated, citrate-depleted and GAP functionalized) p. S2
2. Calculation of the amount of the surface coverage by citrate ions and of the relative amount of GAP per nanoparticle p. S3
3. XPS Analysis of citrate-passivated AuNPs used in this work p. S5
4. Effect of different additives to citrate-depleted AuNPs p. S6
5. Analysis of the evolution of GAP under aerobic condition p. S7
6. Evolution of GAP-functionalized AuNPs under anaerobic conditions p. S8

1. **Thermogravimetric analyses of three different samples (citrate passivated, citrate-depleted and GAP functionalized)**

The % reported in Fig S1 represent the amount of organic material present in the three cases.

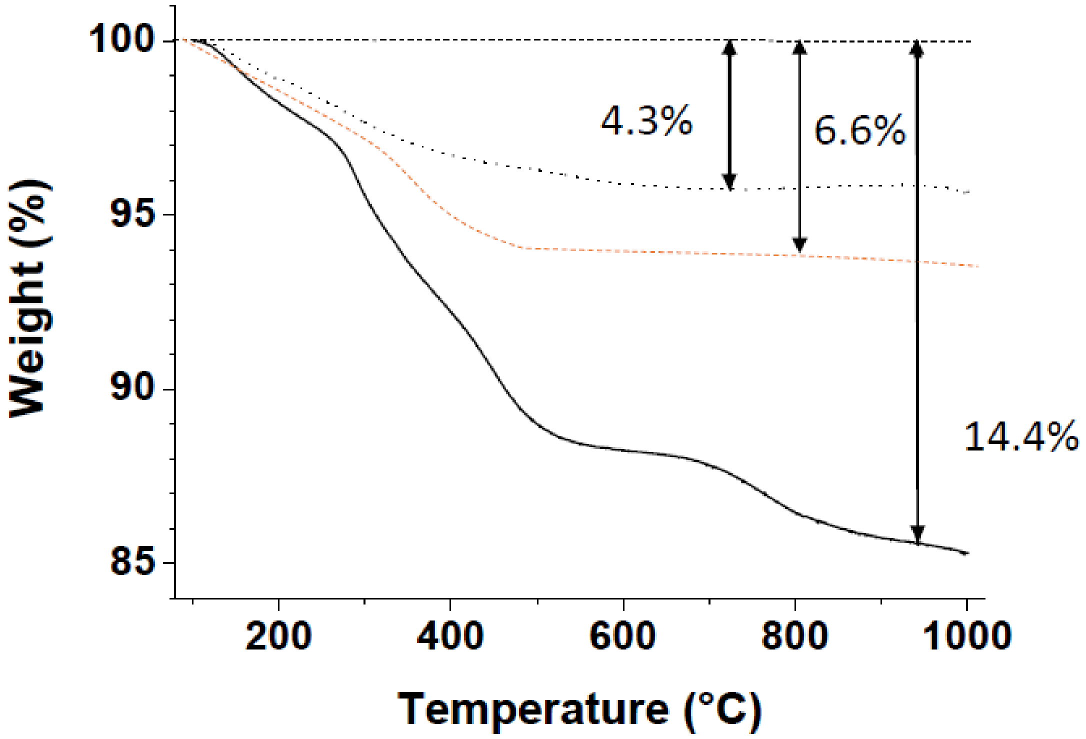

Figure S1. Thermogravimetric analysis (TGA) of the citrate-passivated AuNPS (solid line); citrate-depleted AuNPs with no GAP added (dotted black line); citrate-depleted AuNPs with GAP added (dotted orange line).

2. **Calculation of the amount of the surface coverage by citrate ions and of the relative amount of GAP per nanoparticle**

The amount of surface gold atoms per nanoparticle (n_{out}) was calculated as previously reported in the literature[1] by considering the AuNPs as spherical employing the following formula:

$$n_{out} = \left(\frac{4\pi}{3v_g}\right)[(R-\delta)^3 - (R-\delta-2.38)^3]$$

Where v_g is the average volume of a gold atom (17 Å^3), R is the radius of the nanoparticle and the organic layer, δ is the thickness of the organic monolayer and 2.38 is the thickness of the outer shell of gold atoms on the nanoparticle (see ref.). All previous distances are expressed in Å. Notice that despite the difficulty to obtain a reliable value of the organic layer thickness (δ), R – δ is equal to the radius of the gold core only, measured with high precision from TEM images, being 45 Å in our case (9 nm diameter nanoparticles).

With these numbers, we obtained a number of surface gold atoms per NP equal to 3792. By employing the total volume of the gold sphere and the average volume of a gold atom, we obtained 27800 as the number of gold atoms for a 9 nm nanoparticle. Therefore 13.64% of the total gold is actually on the surface of the nanoparticles.

From the thermogravimetric analysis data, we obtained the mass ratio between gold and organic (in our case only citrate) and by applying the atomic weight of gold (196.96 g/mol) and the molecular weight of citrate (192.12 g/mol as base form) we converted them to molar amounts. The number of citrate molecules per gold surface atom was then obtained by applying the percentage of surface Au atoms obtained above and dividing the resulting amounts. Results are summarized in Table S1.

Table S1. Thermogravimetric analysis of "as-prepared" and washed nanoparticles. 1 mg of nanoparticles taken as an arbitrary value for the calculations

Sample	"As-prepared"	Washed
% Organic	14.37	4.28
mg citrate	0.1437	0.0428
mg Au	0.8563	0.9572
µmol citrate	0.74795	0.22277
µmol Au	4.34758	4.84987
µmol Surface Au	0.591752	0.66148
Citrate / Surface Au	1.26	0.34

Considering the most stable binding mode of citrate to gold as that in which the two oxygen atoms of a carboxylate group bind two gold atoms[2] thus with a 1:2 citrate:gold

coverage ratio, at most 68% of surface Au will be bound to citrate, with still 32% naked and available for binding.

The concentration of nanoparticles in the cuvette was precisely determined from the absorption of the plasmon maximum band. Here we present one example of these calculations. We calculated the extinction coefficient of our nanoparticles by applying the formula obtained by Huo and coworkers.[3] We obtained a value of the extinction coefficient of 1.032×10^{8} $M^{-1}\cdot cm^{-1}$ for nanoparticles of ca. 8.5 nm in diameter. From the absorption of the maximum at 515 nm (0.800) and the extinction coefficient, the concentration of nanoparticles in the cuvette was easily obtained by applying the Lambert-Beer's Law, being 7.8 nM. That value multiplied by number of gold atoms per nanoparticle obtained previously (3792) gives the concentration of surface gold, 29.6 µM. The concentration of residual GAP was estimated to be 5 µM, hence it is six-times smaller than that required to fully passivate the AuNPs surface.

3. XPS Analysis of citrate-passivated AuNPs used in this work

The surface chemical composition of the samples was determined by X-ray Photoemission Spectroscopy. Surface elemental composition (at%) determined by XPS quantitative analysis is reported in Table S1. Au 4f photoemission line (Fig. S2) showed two symmetric peaks attributed to Au $4f^{7/2}$ and Au $4f^{5/2}$ level at binding energies (BE) of 84 eV and 87.7 eV, respectively. The deconvolution of the Au 4f photoemission line showed the presence of two components. The main one, centered at 84 eV, is attributed to metallic gold. The second one, centered at 85.1 eV, is attributed to partially charged $Au^{+\delta}$ species[45] and represents 9% of the gold species.

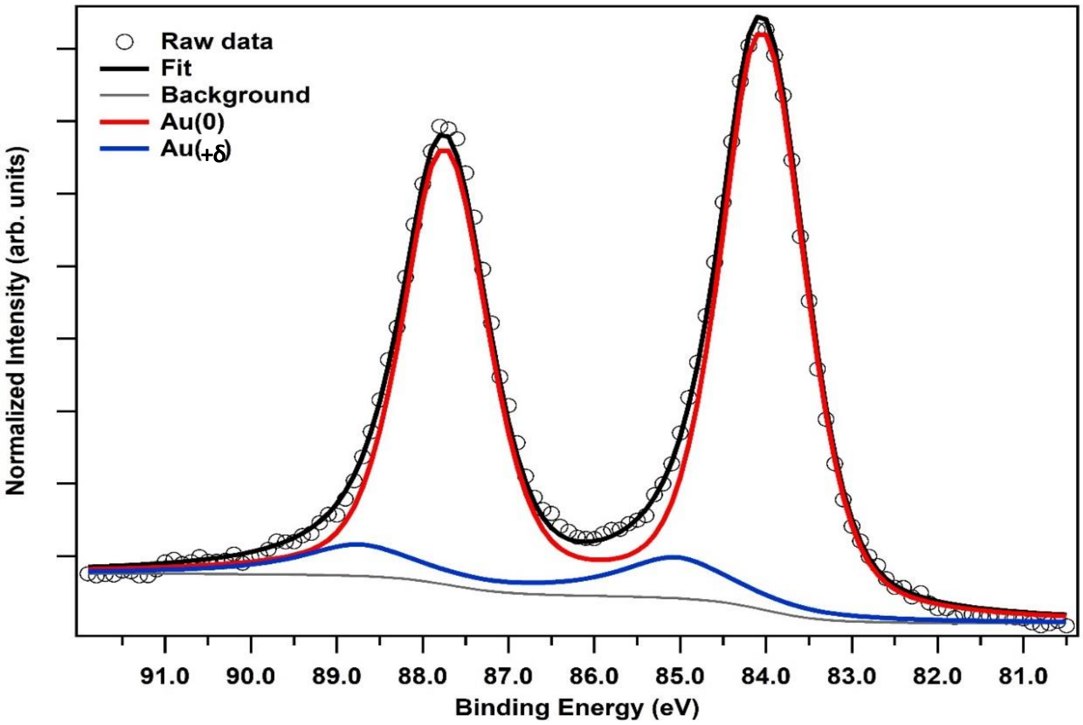

Figure S2. Au 4f photoemission line.

Table S2: Surface elemental composition

**Element**	**Surface elemental composition (at%)**
O	18
C	32
Na	20
Ag	2
Au	28

4. **Effect of different additives to citrate-depleted AuNPs.**

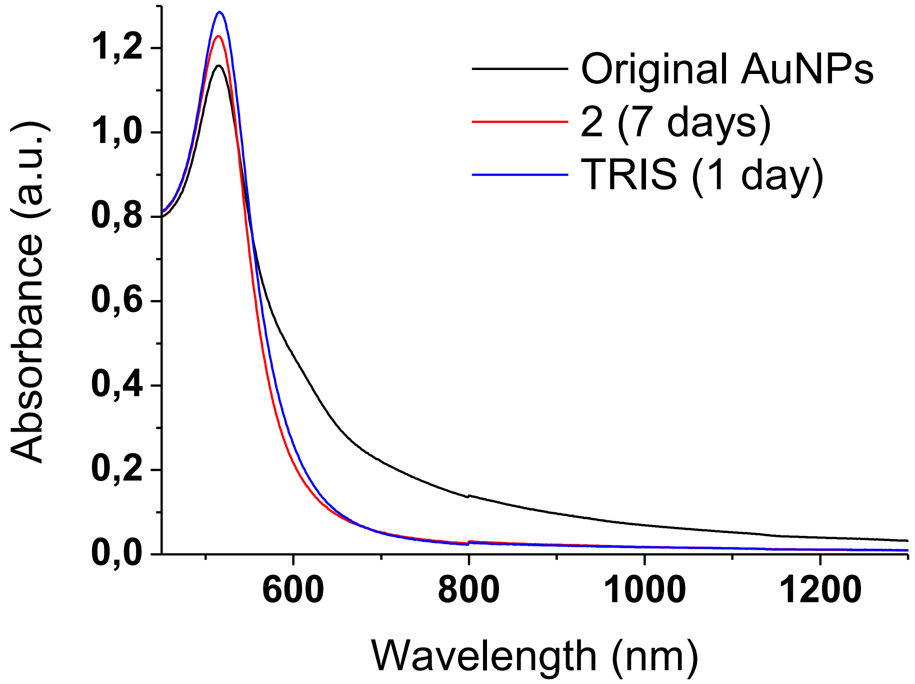

Figure S3. Effect of addition of **2** (incubated for up to 7 days) or TRIS (1 day) to AuNPs. 1. The reference (black) curve refers to the citrate-depleted AuNPs devoid of any additive after 7 days.

5. Analysis of the evolution of GAP under aerobic condition

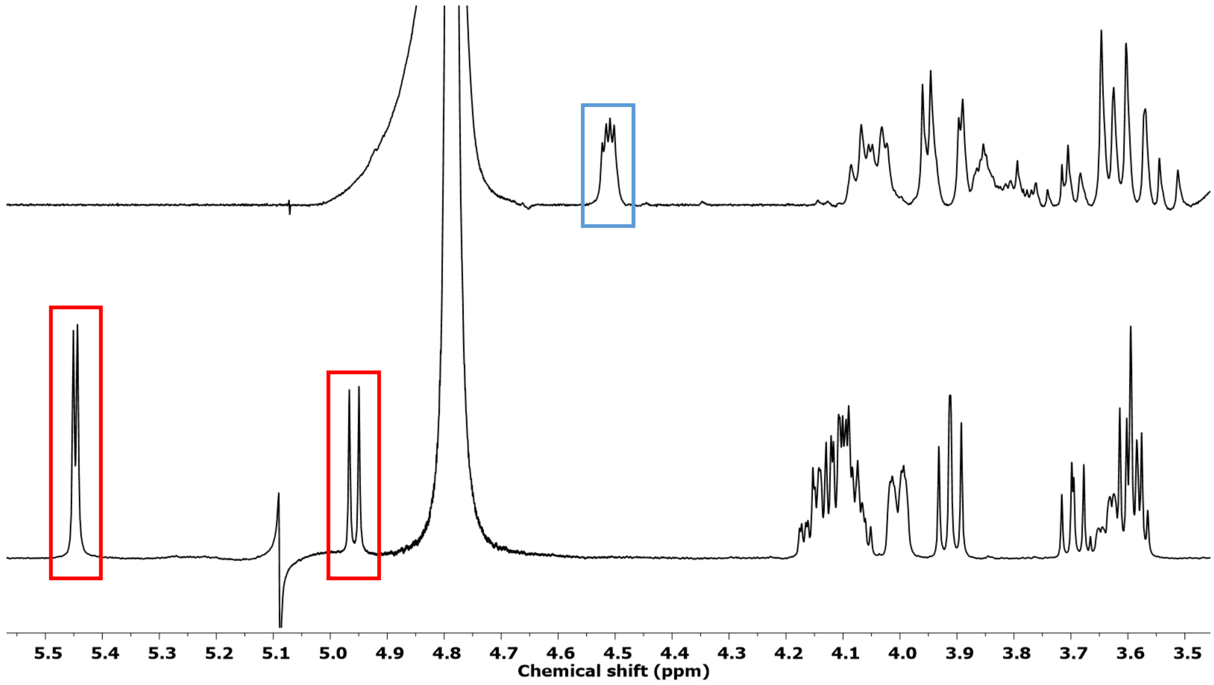

Figure S4. ^{1}H-NMR spectra (D_2O) of GAP (bottom) and the filtrate of a GAP solution in the presence of AuNPs after 2 weeks. Signals corresponding to the α and β anomers of GAP are highlighted with red boxes whereas H-2 of the oxidized form is shown inside a blue box.

6. **Evolution of GAP-functionalized AuNPs under anaerobic conditions.**

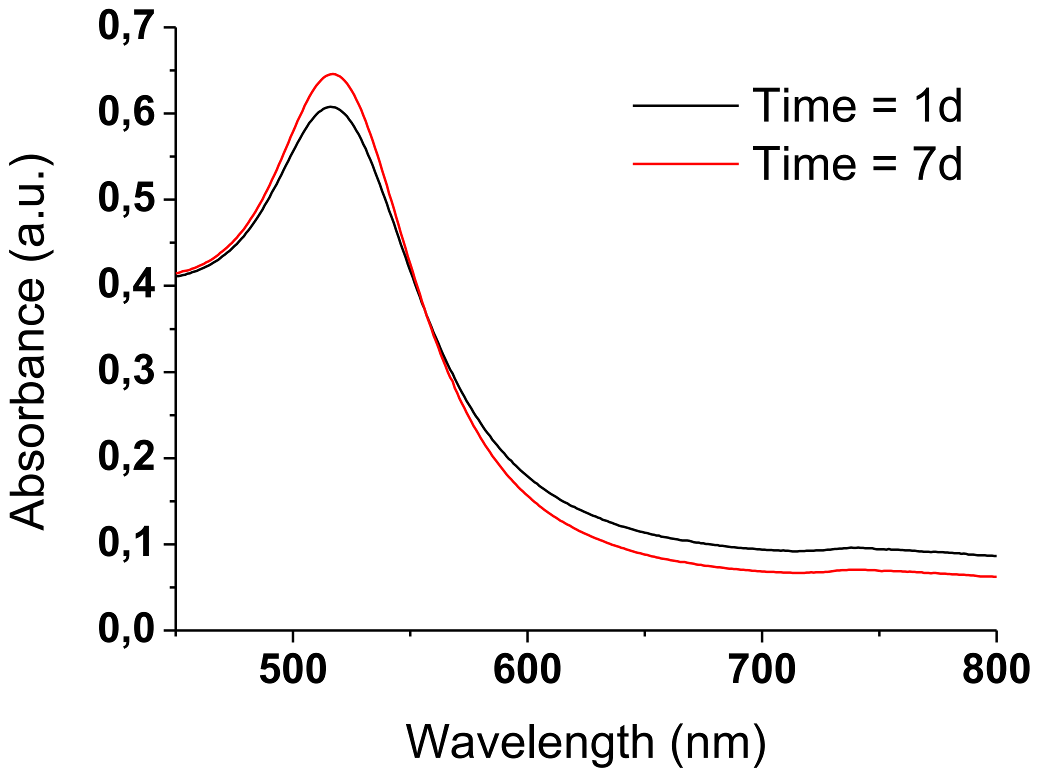

Figure S5. UV-Vis spectra of AuNPs treated with GAP in the absence of O_2 after 2 and 7 days

[1] *J. Phys. Chem.* **1995**, *99*, 7036-7041.

[2] *Nat. Chem.* **2017**, *9*, 890-895.

[3] X. Liu, M. Atwater, J. Wang, Q. Huo, *Colloids Surfaces B Biointerfaces* **2007**, *58*, 3–7.

[4] M. P. Casaletto, A. Longo, A. Martorana, A. Prestianni, A. M. Venezia, Surf. Interface Anal. 2006; 38: 215–218.

[5] Hind Al-Johani, Edy Abou-Hamad, Abdesslem Jedidi, Cory M. Widdifield, Jasmine Viger-Gravel, Shiv Shankar Sangaru, David Gajan, Dalaver H. Anjum, Samy Ould-Chikh, Mohamed Nejib Hedhili, Andrei Gurinov, Michael J. Kelly, Mohamad El Eter, Luigi Cavallo, Lyndon Emsley and Jean-Marie Basset, Nature Chemistry, 9, 890–895 (2017).
